# Iconic Native Culture Cues Inhibit Second Language Production in a Non-immigrant Population: Evidence from Bengali-English Bilinguals

**DOI:** 10.3389/fpsyg.2016.01516

**Published:** 2016-10-05

**Authors:** Kesaban S. Roychoudhuri, Seema G. Prasad, Ramesh K. Mishra

**Affiliations:** ^1^Department of Biochemistry, School of Life Sciences, University of HyderabadHyderabad, India; ^2^Centre for Neural and Cognitive Sciences, University of HyderabadHyderabad, India

**Keywords:** bilingualism, language production, culture cues, parallel language activation

## Abstract

We examined if iconic pictures belonging to one's native culture interfere with second language production in bilinguals in an object naming task. Bengali-English bilinguals named pictures in both L1 and L2 against iconic cultural images representing Bengali culture or neutral images. Participants named in both “Blocked” and “Mixed” language conditions. In both conditions, participants were significantly slower in naming in English when the background was an iconic Bengali culture picture than a neutral image. These data suggest that native language culture cues lead to activation of the L1 lexicon that competed against L2 words creating an interference. These results provide further support to earlier observations where such culture related interference has been observed in bilingual language production. We discuss the results in the context of cultural influence on the psycholinguistic processes in bilingual object naming.

## Introduction

Are bilinguals slower in naming in second language in the presence of images belonging to the native culture? While the study of bilingualism from a cognitive perspective is important for the psycholinguistic understanding of language processing, bilingualism itself can also serve as a good model to study language-culture interaction in the cognitive domain (Kroll and McClain, 2013). Cultural cues such as faces representing one's race or even iconic images of one's culture i.e., the great wall of China for Chinese have shown to disrupt L2 production in Chinese-English bilinguals (Li et al., 2013; Zhang et al., 2013). At the core of this effect lies the fact that bilinguals activate both the languages during object naming in any one language (Jared and Kroll, 2001; Hoshino and Kroll, 2008; Friesen and Jared, 2012; Costa and Sebastián-Gallés, 2014). Native culture cues seem to induce higher activation of the L1 lexicon that interferes with the non-native language production in such bilinguals even when they are living in another culture. Interestingly, these effects are seen in bilinguals that are otherwise good in second language (Zhang et al., 2013). However, this effect has not been replicated in bilinguals in different cultural contexts and with different bilingual experience. In this study, we further examined this issue of native culture's constraining effect during second language production in a group of highly proficient Bengali-English bilinguals in India using the picture naming task.

Bilingual speakers are sensitive to visual cues in the environment that help them control the output language. Culture cues such as faces or other images can exert influence on language selection and production in bilinguals since the bilinguals' language processing mechanism is sensitive to them. Zhang et al. (2013) examined the influence of culture cues (both faces and iconic images) on second language production in Chinese immigrant students living in the US. The authors found higher disfluencies in English speech in such speakers when they were speaking to a Chinese face in a simulated dialogue situation. This has been linked to automatic activation of the native language (L1) lexicon triggered by culture cues which interfered with L2 production. Disruption in English production was also noticed when iconic images were presented in place of faces suggesting the constraining influence of the culture cue. Further, the authors observed that Chinese speakers were faster in literal translation recognition, and they argued that this was because of the heightened activation of L1 lexicon triggered by the culture cues. These speakers also used greater literal translations during object naming in English. This shows that in such a population, even when these participants were staying in an L2 dominant culture for a long time and had appreciable proficiency in the second language, there was disruption in L2 production when they encountered an L1 culture cue. In another study, Li et al. (2013) examined if there was facilitation and interference when there was a match between the language associated with a face cue and the language to be used in naming in a study of Chinese-English bilinguals staying in the US. These speakers were faster in naming when a face cue (Chinese face vs. Caucasian face) was congruent with the language cue. However, there was no disruption in naming in the second language when there were Chinese faces. The differences between these two studies could arise because of differences in design or participants' characteristics (See Li et al., 2013). It is also possible that while one group showed interference during L2 naming in the presence of L1 culture cues, another group did not show because the participants differed in length of their immersion in the L2 dominant context and had different degrees of sensitivity and control settings for L1 and L2. Yang and Yang (2013) have raised the point that the Zhang et al. (2013) study did not particularly control for the language proficiency of the Chinese-English bilinguals. They argue that their lower performance in L2 naming could be because they were not that proficient in L2. In the Zhang et al. (2013) study, the Chinese-English bilinguals had stayed in the US ranging from 3 to 14 months and presumably had low proficiency in L2. In contrast, Li et al. (2013) had taken Chinese-English bilinguals who had acquired L2 early (at an average age of 10.64 years ± 2.59) and were possibly proficient in English.

Is duration of immersion and its context important for such effects? Bilingual's control settings and sensitivity toward L1 has been shown to change with short-term immersion experience in another culture (Kroll et al., 2014). Linck et al. (2009) showed that L2 immersion experience can lead to a weakening of L1 activation which is not seen in classroom learning. Therefore, it is reasonable to assume that depending on the duration of immersion in a non-native culture and the demands of language use, bilingual speakers will show sensitivity to native culture cues to different degrees which may produce facilitation, interference or both during language production. Additionally, while the native culture cue may capture attention, it is the language control setting that modulates any possible interference or facilitation one may see in bilinguals which are further accentuated by the overall language context and their duration of immersion. Therefore, an interesting group of bilinguals to study the cultural influence on language production would be those who have not migrated to another country but have undergone some immersion and shift in their language experience while still living in their native country.

In this study, we examined the constraining effect of native iconic cultural cues on language production in both L1 and L2 in a group of Bengali-English bilinguals. They were students who had been studying at the University of Hyderabad, situated in the Southern state of Telangana for an average of 2 years. Hyderabad is a multi-cultural metropolis and has a large number of people from other Indian provinces. Thus, these students at the time of data collection were not living in their L1 dominant environment (Bengali) but in a university where lingua franca of communication is largely English. It is likely that our population of Bengali-English bilinguals did go through the experience of immersion which should influence their control settings and their sensitivity to the native culture cues. However, it is important to note that this situation is different from when bilinguals migrate to another country in which case the status of L1 would be significantly diminished. These bilinguals spoke both English and Bengali, often switching between the two. The lingua-franca of the university being English, many of the bilinguals showed excellent proficiency in English and were dominant in this language^1^. Therefore, expecting an influence of L1 culture cue on their L2 planning and production may not be warranted as such. Thus, it will be interesting to see if native culture cues still influence language production in such a bilingual sample.

In this sense, our study uniquely tests a group of bilinguals, who while living in their country have undergone a shift in language use pattern because of change in language environment which has not been studied before. Following the findings of the earlier studies on Chinese-English bilinguals, we would expect that these bilinguals would also show sensitivity to L1 culture cues during L2 naming which should result in interference. We selected pictures that represented Bengali culture in a popular way (i.e., which every Bengali can identify as being linked to their culture). We hypothesized that if cultural cues such as faces can lead to interference in L2 naming, then these iconic pictures should also show similar effects. Thus, the design of this study allowed us to test whether culture cues related to the native language would inhibit L2 production in highly proficient bilinguals who are otherwise known to activate both the languages in parallel. The culture cues were presented in the background throughout the trials thus making them completely irrelevant to the main task. We chose this design because we speculated that presenting the culture images for a short while before the participants named the object would lead to explicit association of the corresponding language with the culture cue. In one context (“Blocked” context), participants named the pictures either in English or Bengali in blocks while the culture cues were randomized. This was to see if culture cues would influence naming in L2 (interference) and L1 (facilitation) when participants do not need to switch between languages.

In the second context, participants were randomly cued to name in a particular language (“Mixed” context) on each trial. Thus, the “Mixed” context included switching between the languages. It has been shown that language naming induces different control settings when naming language is blocked compared to when it is mixed since it involves random switching between languages (Misra et al., 2012). Participants may exercise a long-term control setting when naming is blocked where they name objects in one language for a long time. Similarly, when speakers have to switch between alternatives on every trial, they may bring in more transient control settings. Therefore, it is important to examine the interference induced by the culture cue and how it influences L1 and L2 naming in these two situations. This is particularly interesting since the culture cue may provide an additional cue for language selection particularly for the mixed block by biasing the level of lexical activation for one language, and this may result in increased conflict influencing latency of speech onset. Thus, we tested naming in both blocked and mixed contexts.

We predicted that, if bilinguals are in general sensitive to native culture cues and language context, then we should observe interference in English naming in Bengali-English bilinguals in the presence of native culture cues. We also expected the native culture cues to facilitate naming in Bengali (L1) following the observations of Li et al. (2013). It would be further interesting to see how the culture cues modulate the trial-by-trial switching of languages in the mixed block. Language switching in the mixed blocks has been known to produce switch costs which are asymmetric (Meuter and Allport, 1999). This primarily results from the inhibition of a more dominant L1 while naming in L2. However, it has been argued that proficiency plays a major role in determining asymmetry in switch costs. It has been observed that bilinguals highly proficient in L1 and L2 do not show asymmetric switch costs (Costa and Santesteban, 2004; see Bobb and Wodniecka, 2013 for a review). Since the Bengali—English bilinguals in our study were highly proficient in L2, it is likely that asymmetric switch costs will not be observed with this population in the neutral cue condition. However, we predict that in the presence of a culture cue L1 activation would be boosted resulting in easier switching from L2 to L1 as opposed to switching from L1 to L2. Thus, we would expect participants to incur lower switch costs while switching from L2 to L1 in the presence of a native culture cue as opposed to a neutral cue.

## Methods

### Participants

Forty-eight Bengali-English bilinguals^2^ (20 females and 28 males, Mean age = 23.5 years, *SD* = 2.02 years) participated in the main experiment. All the participants had Bengali as the native language and had acquired English as a second language through formal education at school. The mean age of acquisition of English was 5.28 years (*SD* = 2.12 years). All the participants were staying at the University of Hyderabad for 2 years preceding to which they were in their native province (i.e., West Bengal). All participants provided written consent for their participation. The study was approved by the Institutional Ethics Committee of University of Hyderabad.

Participants' proficiency in L1 (Bengali) and L2 (English) was assessed using a language background questionnaire that had questions on the native language, languages known, the age of acquisition of L1 and L2, percentage of time exposed currently to L1 and L2, and daily usage of L1 and L2 in both work and non-work related activities (Table 1). They also provided self-rating for proficiency in both the languages (L1 and L2) for reading, speaking fluency, and listening ability on a ten-point scale ranging from *poor* (1) to *excellent* (10). There was no significant difference between the L1 and L2 ratings, *t*_(1, 47)_ = 0.66, *p* = 0.5. Participants with higher L1 self-report score were considered to be dominant in Bengali and participants with higher L2 self-report score were considered to be English dominant. Twenty-two of the participants were dominant in Bengali (L1 self-report score = 9.5, *SD* = 0.51, L2 self-report score = 8.02, *SD* = 0.49). Twenty-six of the participants were dominant in English (L1 self-report score = 7.37, *SD* = 1.00, L2 self-report score = 9, *SD* = 0.76).

**Table 1:** **Language proficiency and demographic details of the participants**.

	**Mean (*SD*)**	**Range**
Age (in years)	23.54 (2.02)	18–28
Age of acquisition of L2 (years)	5.28 (2.12)	2–12
Years of education in L2	13.14 (5.84)	4–20
Vocabulary test (L2)	52.6% (11.53)	27–73
Semantic fluency (L1)	13.93 (2.5)	6.5–23.5
Semantic fluency (L2)^†^	11.45 (3.86)	5–22
Score on self-report questionnaire (L1)	8.37 (1.34)	5.6–10
Score on self-report questionnaire (L2)	8.55 (0.81)	7–10

† *Marginally significant differences between L1 and L2 fluency scores, p < 0.1*.

All the participants completed an online vocabulary test (WordORnot, Center for Reading Research, Ghent University) which was administered to test their proficiency in L2. The test required the participants to judge strings of English letters as a “word” or a “non-word.” Participants were instructed to maintain speed and accuracy in this task. The total score was the difference between the percentage of correct and incorrect responses (Table 1). We also administered semantic fluency tests in both Bengali and English, where participants were asked to generate as many names as they could of everyday objects in 1 min. In Bengali, they produced names of vegetables and birds and for English, they were asked to generate names of animals and fruits. Different categories were used for both the languages to avoid the confounding effect of having recalled words of a particular category recently while naming the second time. The average number of words produced per language per minute was calculated for analysis. There was a marginally significant difference in the semantic fluency scores between Bengali (*M* = 14.19, *SD* = 3.02) and English (*M* = 13.2, *SD* = 4.06), *t*_(1, 47)_ = 1.81, *p* = 0.07. However, participants dominant in Bengali performed significantly, *t*_(1, 21)_ = 6.27, *p* < 0.001 better on the L1 fluency task (*M* = 14.3, *SD* = 2.97) compared to the L2 fluency task (*M* = 11.25, *SD* = 3.7).

### Material and stimuli

Six iconic images representing the cultural heritage of Bengal were selected as culture cues (Appendix 1 in Supplementary Material). These images measured 960 × 720 pixels each. The images were selected from freely available pictures from image repertoires such as Google images. It was made sure that the selected cultural cues would be easily recognized by all Bengali participants. Two images of simple textures of sizes comparable with culture cues were selected as neutral cues (Appendix 2 in Supplementary Material). Ten Bengali-English bilinguals, who were students at the University of Hyderabad were asked to rate these images on a scale of 1–5 (1, not related to Bengali culture; 5, highly related to Bengali culture). Participants rated the images of Goddess Durga and Howrah Bridge with scores of 4.8 and 4.7 which were then selected to be used as stimuli. The neutral images received a rating of 2.2 (*SD* = 1.13) and 2.3 (*SD* = 1.15), respectively. The bilinguals who rated the images did not participate in the main experiment. One hundred line drawings measuring 300 × 300 pixels were selected to be used as stimuli in the naming task (Supplementary Figure 1). These pictures included line drawings of common objects. Pictures with phonetically similar onset in Bengali and English as well as pictures having multiple names in Bengali and English were not considered. Fourteen Bengali-English bilinguals (who did not participate in the main experiment) were asked to rate the pictures in both the languages. Raters were asked to rate their level of agreement between the names and the pictures on a scale of 1–5 (1, lowest agreement; 5, highest agreement). They also rated the frequency of use of words in both the languages (1, lowest frequency; 5, highest frequency). Pictures that received more than a score of 4 (out of 5) in name agreement and frequency of use in both Bengali and English were selected. There was no significant difference [*t*_(1, 13)_ = 0.402, *p* = 0.694] in the agreement ratings for Bengali (*M* = 4.63, *SD* = 0.29) and English names (*M* = 4.57, *SD* = 0.47). Frequency of use of Bengali (*M* = 2.99, *SD* = 0.36) and English (*M* = 2.91, *SD* = 0.38) words did not differ significantly [*t*_(1, 13)_ = 0.46, *p* = 0.65].

### Procedure

**Figure 1:**
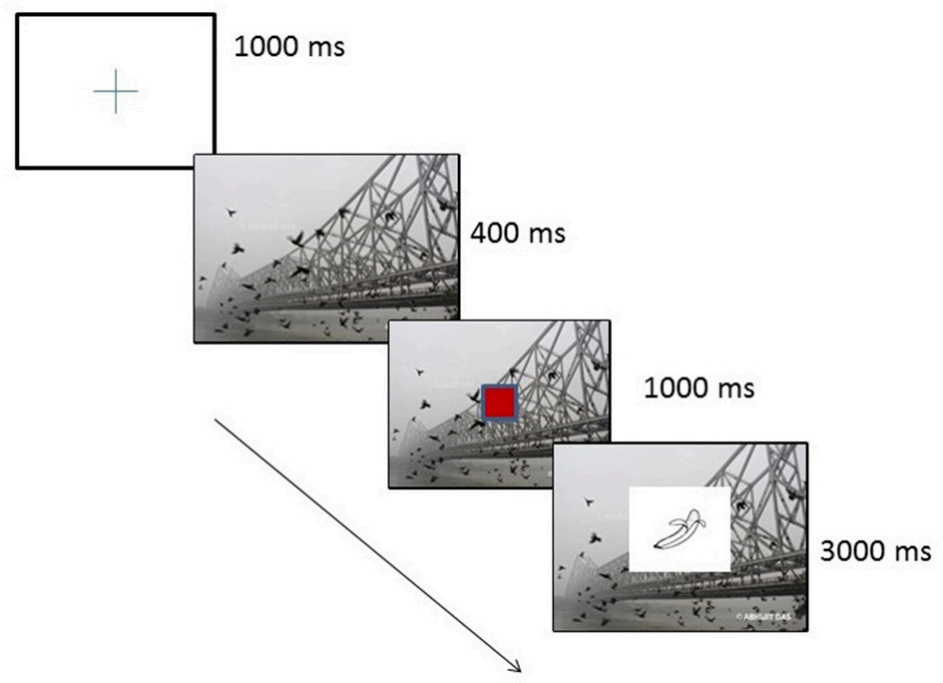
**Sample trial showing sequence of events**.

Participants were seated at a distance of 60 cm from an LCD monitor with 1366 × 768-pixel resolution and with a screen refresh rate of 60 Hz. A microphone connected to the computer recorded the speech. Before the experiment, each participant was shown all the pictures used as stimuli and their names (in L1 and L2). Participants were told that they would see the same pictures in the main experiment and would be asked to name them in either English or Bengali according to the language cue that was given before each block. They were not told anything about the background image (culture cue or neutral image) which changed randomly from trial to trial. They were told to name the objects fast and accurately. Each trial started with a fixation cross that appeared at the center of the screen for 1000 ms followed by the culture/neutral cue which stayed in the background for the rest of the trial (See Figure 1 for illustration of a sample trial). After 400 ms, the language cue appeared for 1000 ms. The language cues were red or green boxes measuring 40 × 40 pixels. A picture was then presented for a maximum time of 3000 ms. The participants were instructed to name the picture in L1 (Bengali) if the language cue was a “Red Box” and in L2 if the language cue was “Green box.” This mapping between the color of the cue and the language to be spoken was counterbalanced across participants. An inter-trial interval of 1000 ms was given after every trial.

### Design

The experiment had a total of 400 trials divided into two contexts: “Blocked” and “Mixed.” Both the contexts had the same set of trials. Each context had 200 trials and was further divided into four blocks. The 100 line drawings were repeated twice in each context. Every line drawing was named once in Bengali and once in English in each context. The “Blocked” and the “Mixed context” were done within a gap of 1–5 days. The order of the contexts was roughly counterbalanced across participants. In the “Blocked” context, each of the four blocks consisted of a single language cue (indicating either L1 or L2) and the background cues were presented in random order. Thus, there were two blocks in which pictures were to be named in L1 and two blocks in L2. In each block, the trials were equally divided between the two types of culture cues and two types of neutral cues. In the “Mixed” context, language cues were also randomized for each participant. The order of the trials in each block, as well as the order of the blocks, was completely randomized for all participants. Each participant was given 10 practice trials in each context. A single break was given halfway through a context and the experiment resumed automatically after 5 min. For analysis, we compared L1 and L2 naming in the presence of the culture cues vs. neutral cues.

## Results

Naming latency was computed by the software as the time at which the voice-key was triggered after the display of the line drawing. 3.6% of trials in which the object was named incorrectly or where the voice key was triggered due to non-verbal sounds were excluded from analysis. The trials in which the naming was in the language opposite to the cue were coded as “language errors.” There were only 1.1% of such trials. Hence, no error analysis was done, and they were excluded from further analysis. Trials with response times < 250 and >2000 ms were filtered and discarded. There were a total of 7.1% of such trials. Data of two participants were discarded from further analysis due to faulty recording in the “Blocked” context. Three subjects' data was discarded because of a high percentage of data loss in the “Mixed” context. Two subjects' data was discarded as the average naming latency was >2 *SD* from the total average in either of the contexts.

### Naming latency

**Figure 2:**
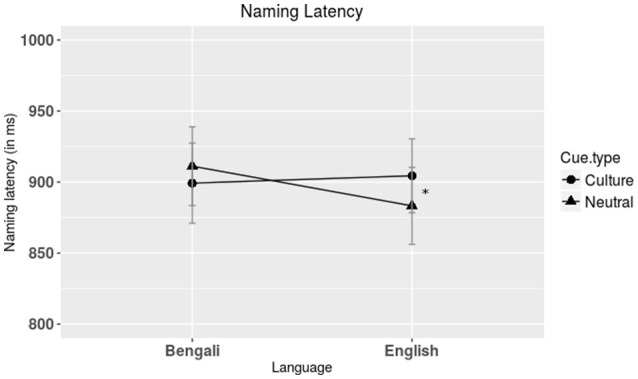
**Plot showing response times for L1 and L2 naming against cultural and neutral cues**. The culture cue facilitated L1 naming and inhibited L2 naming, compared to neutral cue. Note: ^*^p < 0.01.

Repeated measures ANOVA was performed on the remaining trials with Response time as the dependent measure and context (blocked, mixed), cue type (cultural, neutral), and language (Bengali, English) as factors (See Table 2 for condition-wise RTs). ANOVA was performed both by subjects (*F*_*1*_) and by items (*F*_*2*_) (see Appendix 3 in Supplementary Material for the results in a table form). There was a no significant effect of context by subjects, *F*_*1*(1, 40)_ = 0.07, *p* = 0.79, η^2^ = 0.002 but it was significant by item, *F*_*2*(1, 98)_ = 8.13, *p* = 0.005, η^2^ = 0.08. There was a significant interaction between cue type and language by subjects, *F*_*1*(1, 40)_ = 6.44, *p* = 0.01, η^2^ = 0.14 and by items, *F*_*2*(1, 98)_ = 4.35, *p* = 0.04, η^2^ = 0.04 (Figure 2). Pairwise comparisons showed that participants were slower (*p* = 0.009) in naming in L2 (English) in the presence of a culture cue (*M* = 911.21 ms, *SE* = 27.7) compared to a neutral cue (*M* = 883.27 ms, *SE* = 27.09) Also, participants were faster naming in L1 when a cultural cue was present in the background (*M* = 899.25 ms, *SE* = 28.17) as opposed to when a neutral cue was present (*M* = 911.21 ms, *SE* = 27.7). However, this effect did not reach statistical significance (*p* = 0.21). There was no main effect of cue or language by subjects, *F*_*1*(1, 40)_ = 0.69, *p* = 0.41, η^2^ = 0.02 and *F*_*1*(1, 40)_ = 1.65, *p* = 0.21, η^2^ = 0.04, respectively. Similarly, item wise analysis did not reveal any significant effects of cue *F*_*2*(1, 98)_ = 0.004, *p* = 0.95, η^2^ < 0.001. Language had a significant effect in the item-wise analysis, *F*_*2*(1, 98)_ = 4.84, *p* = 0.03, η^2^ = 0.05. The interaction between context and cue type was not significant by subject, *F*_*1*(1, 40)_ = 0.64, *p* = 0.43, η^2^ = 0.02 or by item, *F*_*2*(1, 98)_ = 0.44, *p* = 0.51, η^2^ = 0.01. The interaction between context and language was not significant by subject, *F*_*1*(1, 40)_ = 2.25, *p* = 0.14, η^2^ = 0.05 but significant by item *F*_*2*(1, 98)_ = 5.9, *p* = 0.02, η^2^ = 0.06. The three-way interaction between context, cue type, and language did not turn out to be significant, *F*_*1*(1, 40)_ = 0.22, *p* = 0.64, η^2^ = 0.005, and *F*_*2*(1, 98)_ = 0.02, *p* = 0.89, η^2^ < 0.001.

**Table 2:** **Naming latencies for L1 and L2 naming (in ms)**.

		**Combined (*****n*** = **41)**		**“Blocked” first (*****n*** = **23)**		**“Mixed” first (*****n*** = **18)**	
		**L1 Mean (*SD*)**	**L2 Mean (*SD*)**	**L1 Mean (*SD*)**	**L2 Mean (*SD*)**	**L1 Mean (*SD*)**	**L2 Mean (*SD*)**
“Blocked” context	Culture cue	904.86 (167.46)	901.46 (156.51)	833.36 (173.20)	835.08 (147.03)	996.21 (137.75)	986.28 (136.55)
	Neutral cue	923.26 (175.58)	881.56 (166.82)	873.85 (176.51)	822.03 (155.53)	986.41 (168.07)	957.63 (133.3)
“Mixed” context	Culture cue	893.64 (226.35)	907.44 (207.69)	727.98 (106.81)	755.34 (126.82)	1105.32 (106.86)	1101.79 (77.91)
	Neutral cue	899.16 (229.21)	884.97 (220.29)	742.42 (102.86)	720.8 (152.49)	1099.43 (106.52)	1094.76 (90.13)

“*Blocked” first refers to the participants who participated in the “Blocked” context first. Similarly, “Mixed” first refers to participants who performed the “Mixed” context first. We thank an anonymous reviewer for suggesting to break down the means by block order*.

To examine if the interference observed in L2 naming varied on blocks of trials, repeated measures ANOVA was performed on naming latencies in the “Mixed” context^3^ with block (1, 2, 3, 4), cue type (cultural, neutral), and language (Bengali, English) as factors. The main effect of block was not significant, *F*_(3, 120)_ = 1.14, *p* = 0.33, η^2^ = 0.03.

### Switch costs and mixing costs

**Figure 3:**
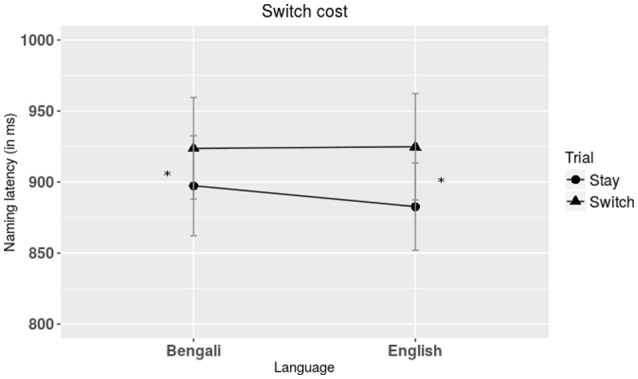
**Plot showing response times for L1 and L2 naming during stay and switch trials**. Symmetric switch costs were observed during switching between the two languages. Note: ^*^p < 0.01.

We calculated switch and mixing costs for naming. Switch costs for “Mixed” context was defined as the difference in latency between “stay” and “switch” trials (Figure 3). Paired *t*-tests were performed to ascertain that there was a main effect of trial type, *t*_(1, 40)_ = 3.89, *p* < 0.001, *d* = 0.61, that is stay responses (*M* = 890.1 ms, *SD* = 206.4) were faster than responses on switch trials (*M* = 925 ms, *SD* = 232.5). Repeated measures ANOVA was then performed on switch costs with cue type (cultural, neutral) and language (Bengali, English) as factors. There was no main effect of cue type or language, *F*_(1, 40)_ = 0.69, *p* = 0.41, η^2^ = 0.02 and *F*_(1, 40)_ = 0.70, *p* = 0.40, η^2^ = 0.02, respectively. The interaction between cue type and language was not significant either, *F*_(1, 40)_ = 2.09, *p* = 0.16, η^2^ = 0.05.

Mixing costs were calculated for each language by comparing the response times for “Blocked” and the “Mixed” contexts under different cue conditions. For example, L1 mixing cost = Response time for L1 naming (on stay trials) in the “Mixed” context—Response time for L1 naming in the “Blocked” context. Repeated measures ANOVA was performed with context (blocked, mixed), language (Bengali, English), and cue type (cultural, neutral) as factors to examine mixing costs. Main effect of context was not significant, *F*_(1, 40)_ = 0.32, *p* = 0.57, η^2^ = 0.01. Main effect of language was marginally significant, *F*_(1, 40)_ = 3.03, *p* = 0.09, η^2^ = 0.07. Participants were faster naming in English (*M* = 887.12, *SE* = 25.16) compared to Bengali (*M* = 905.74, *SE* = 27.96). Neither the main effect of cue type, *F*_(1, 40)_ = 2.24, *p* = 0.14, η^2^ = 0.05 nor the interaction between context and cue type, *F*_(1, 40)_ = 2.49, *p* = 0.12, η^2^ = 0.06 was significant (See Appendix 3 in Supplementary Material for the complete results).

## Discussion

In this study, we tested if native language culture cues influenced object naming in L1 and L2 in a Bengali-English bilingual population which has been living away from their native culture. Our results show that Bengali-English bilinguals faced interference during object naming in English in the presence of Bengali culture cues. Also, L1 culture cues facilitated naming in L1 compared to baseline (neutral cue). However, this effect did not reach statistical significance. This pattern was observed for both “Blocked” and “Mixed” contexts. There was no difference in naming latencies between mixed and blocked trials. We also computed switch and mixing costs for L1 and L2 naming. No significant costs were incurred due to mixing of languages as seen from non-significant mixing costs. We did not find a significant influence of Cue type on mixing costs for both L1 and L2 naming. The characteristic asymmetric switch costs for L1 and L2 naming was not observed. Switch costs were not modulated by the type of background cue either, although the interaction between language and cue type appeared to be trending toward marginal significance (*F* > 1). However, it is difficult to determine in our current study if the asymmetric switch costs were due to the balanced nature of the participants or the because of the culture cues themselves. Several studies in the past have failed to observe the asymmetry in switch costs (Christoffels et al., 2007; Verhoef et al., 2009, 2010; Prior and Gollan, 2011; Tarlowski et al., 2013). Bobb and Wodniecka (2013) have discussed at length the source of symmetric switch costs. They suggest that symmetric switch costs could arise out of a complex interaction between participant characteristics (such as their L2 proficiency) and the inhibitory control processes involved in language production. Although, the reasons for observing symmetric switch cost are not clear from the literature, there seems to be a definitive link between language proficiency and switch cost. For instance, it has been shown there is a negative correlation between L2 proficiency and asymmetrical switch costs (Filippi et al., 2014). Our participants displayed a high level of proficiency in L2 (as indicated by high scores on vocabulary and verbal fluency test as well as on the self-report questionnaire). Thus, it is possible that high L2 proficiency of our participants lead to symmetrical costs during switching between L1 and L2. To test this, we performed a correlational analysis between asymmetric switch costs (calculated as L2 switch cost—L1 switch cost) and L2 self-reported proficiency, following the procedure of Filippi et al. (2014). We expected a negative correlation as high L2 proficient bilinguals should show less asymmetry. We did find a negative correlation, *r*_(41)_ = −0.01, *p* = 0.9, but the effect was not reliable. This could be because all our participants were highly proficient in L2. The correlational analysis is useful when there is a variance in the sample. As there was no distribution of L2 proficiency in our sample, it may have caused the statistics to be unreliable.

The results from this study extend and shed further light on the influence of the native culture cues resulting in interference during L2 production (Li et al., 2013; Zhang et al., 2013). Our results show that even bilinguals with good proficiency and dominance in L2 show interference when they have to name objects against cultural cues belonging to native language. This could be because of native cues leading to activation of L1 words which then competed with L2 words during production. We have replicated the findings of Zhang et al. (2013) showing that bilinguals face interference in second language production in the presence of native culture cues. Our data also suggests that native culture cues could facilitate naming in that language as it has been observed by Li et al. (2013), although this result was not supported statistically. The results suggest that bilinguals remain sensitive to their native culture cues and show its influence on their speech production.

Hartsuiker (2015) reviewing work on the issue of culture/visual cues and language co-activation proposes that it is still not clear if there is just interference (as in Zhang et al., 2013), facilitation (Li et al., 2013) or both facilitation and interference in such tasks. This has been partly the situation because Zhang et al. (2013) did not check the influence of English primes on English production. Whereas, Li et al. (2013) did examine the impact on both languages and observed facilitation when the cue was congruent with the language to be used for naming. We did not use any English culture prime as well. We used only Bengali culture cues and observed interference but non-significant facilitation. Unlike previous studies, we used the object naming task and found the effects when naming was blocked and mixed. Further, most previous studies examining the influence of native culture cues on language production have used faces symbolic of native cultures as visual cues (except for Zhang et al., 2013). Li et al. (2013) base their idea on the theory of “interactive alignment” (Pickering and Garrod, 2004) which proposes that interlocutors adapt to each others' language patterns in order to make communication effective. Thus, they suggest that inferred knowledge about interlocutors (in this case, through the face cues) can influence language production. However, the culture cues in our study did not represent any speaker and thus were not processed actively. Thus, our study is novel in this aspect as we have shown that even subtle influence of visual cues presented in the background can affect language production.

We wondered if the use of the picture of a very popular Goddess “Durga” in any way influenced our results? We performed additional analysis on Response times with type of cue (Goddess Durga, Howrah bridge, Neutral cue 1, Neutral cue 2) as factors. This was done to examine whether there were differences in the extent to which the two types of culture cues (Goddess Durga and Howrah bridge) affected L2 naming. We found that participants were significantly slower in naming in the presence of the image of Goddess Durga compared to Howrah bridge. It is possible that the presence of the image of a Goddess induced anxiety in the participants (Toburen and Meier, 2010), affecting their task performance. The image of Goddess Durga was also visually more complex than the image of Howrah bridge. This could have have also lead to the differences in the response times to the two images. However, L2 naming in the presence of both cultural cues differed significantly compared to the two neutral images. But the overall slowing down in the presence of “Goddess Durga” could be the reason we didn't observe significant facilitation in L1 naming. Thus, it appears that different types of cultural cues could influence language selection differently depending on their salient values and importance. Another possible limitation of our study was the difference in visual salience between the culture cues and the neutral images. Culture cues were more salient due to the complex visual features (face and attire of Goddess Durga, for example) as compared to the neutral images (homogeneous textures of a single color). We did not match the pictures in terms of visual salience which might have affected our results. However, there was no main effect of cue type on naming latency. That is, there were no differences in participants' naming latency in the presence of culture cue vs. neutral cue. This suggests that the culture cues may not have had any additional effect on participants' responses due to their visual features. Nonetheless, future studies should consider controlling for the visual complexity of background cues used in such experiments.

While these results show the modulatory influence of culture cues on language production, they also show that high language proficiency in L2 does not lead to suppression of L1. Inspite of the participants being highly proficient in L2, we observed that L1 culture cues interfered with L2 production. The link between native culture and corresponding lexicons appears to be strong and is automatically activated. This effect probably varies with regard to the types of bilinguals and the language environment. In contrast to earlier studies with Chinese-English bilinguals, our speakers did not fully reside in a L2 dominant context. They still used their native language on a daily basis although some of them showed dominance in L2. Their sensitivity to L1 culture cues remained strong when they switched language during naming. Thus, the interference observed was irrespective of the naming context. Future studies on this issue should examine such bilinguals while they reside in their native culture.

In sum, these results suggest that native culture symbolic cues interfere in second language production in bilinguals. We show here that such effects are also to be seen in non-immigrant bilinguals. Our results suggest that short immersion may not be enough to modulate the control settings for L1 that is required for inhibition during L2 production. The language proficiency and dominance of the participants would play a crucial role in language regulation with respect to a culture cue, especially in terms of language switching. Further, we assume that long-term immersion in a non-native culture should lead to better control of interference from L1 during L2 production. Future studies should also examine early and simultaneous bilinguals to see if language background plays any role in these effects.

## Author contributions

KR, SP, and RM conceptualized and designed the study. KR collected the data. KR and SP analyzed the data. KR, SP, and RM interpreted the results. KR, SP, and RM were involved in the drafting and the final approval of the manuscript.

### Conflict of interest statement

The authors declare that the research was conducted in the absence of any commercial or financial relationships that could be construed as a potential conflict of interest.

## Footnotes

^1^It is to be noted that although all participants had a high level of proficiency in L2, closer inspection of the self-report scores revealed that some of them were dominant in Bengali and some in English. See p. 9 for more details.

^2^Data from 28 bilinguals was collected initially. Additional data collection of twenty bilinguals was done during first revision.

^3^In the “Blocked” context, there was no fixed ordering of the blocks. They were randomized and hence there were 24 possible ways in which the blocks were presented. Hence, the block-wise analysis was not performed as a sequential block effect is less likely to be operating in such a scenario.

## References

[B1] Bobb S. C. Wodniecka Z. (2013). Language switching in picture naming: what asymmetric switch costs (do not) tell us about inhibition in bilingual speech planning. J. Cogn. Psychol. 25, 568–585. 10.1080/20445911.2013.792822

[B2] Christoffels I. K. Firk C. Schiller N. O. (2007). Bilingual language control: an event-related brain potential study. Brain Res. 1147, 192–208. 10.1016/j.brainres.2007.01.137 17391649

[B3] Costa A. Santesteban M. (2004). Lexical access in bilingual speech production: Evidence from language switching in highly proficient bilinguals and L2 learners. J. Mem. Lang. 50, 491–511.

[B4] Costa A. Sebastián-Gallés N. (2014). How does the bilingual experience sculpt the brain? Nat. Rev. Neurosci. 15, 336–345. 10.1038/nrn3709 24739788 PMC4295724

[B5] Filippi R. Karaminis T. Thomas M. S. (2014). Language switching in bilingual production: empirical data and computational modelling. Bilingualism Lang. Cogn. 17, 294–315. 10.1017/S1366728913000485

[B6] Friesen D. C. Jared D. (2012). Cross-language phonological activation of meaning: evidence from category verification. Bilingualism Lang. Cogn. 15, 145–156. 10.1017/S1366728910000489

[B7] Hartsuiker R. J. (2015). Visual cues for language selection in bilinguals, in Attention and Vision in Language Processing, eds Mishra R. K. Srinivasan N. Huettig F. (Springer India), 129–145.

[B8] Hoshino N. Kroll J. F. (2008). Cognate effects in picture naming: does cross-language activation survive a change of script? Cognition 106, 501–511. 10.1016/j.cognition.2007.02.001 17367774

[B9] Jared D. Kroll J. F. (2001). Do bilinguals activate phonological representations in one or both of their languages when naming words? J. Mem. Lang. 44, 2–31. 10.1006/jmla.2000.274727609455

[B10] Kroll J. F. Bobb S. C. Hoshino N. (2014). Two languages in mind bilingualism as a tool to investigate language, cognition, and the brain. Curr. Dir. Psychol. Sci. 23, 159–163. 10.1177/0963721414528511 25309055 PMC4191972

[B11] Kroll J. F. McClain R. (2013). What bilinguals tell us about culture, cognition, and language? Proc. Natl. Acad. Sci. U.S.A. 110, 11219–11220. 10.1073/pnas.1309472110 23784774 PMC3710793

[B12] Li Y. Yang J. Scherf K. S. Li P. (2013). Two faces, two languages: an fMRI study of bilingual picture naming. Brain Lang. 127, 452–462. 10.1016/j.bandl.2013.09.005 24129199

[B13] Linck J. A. Kroll J. F. Sunderman G. (2009). Losing access to the native language while immersed in a second language: evidence for the role of inhibition in second-language learning. Psychol. Sci. 20, 1507–1515. 10.1111/j.1467-9280.2009.02480.x 19906121 PMC2858781

[B14] Meuter R. F. Allport A. (1999). Bilingual language switching in naming: asymmetrical costs of language selection. J. Mem. Lang. 40, 25–40. 10.1006/jmla.1998.2602

[B15] Misra M. Guo T. Bobb S. C. Kroll J. F. (2012). When bilinguals choose a single word to speak: electrophysiological evidence for inhibition of the native language. J. Mem. Lang. 67, 224–237. 10.1016/j.jml.2012.05.001 24222718 PMC3820915

[B16] Pickering M. J. Garrod S. (2004). Toward a mechanistic psychology of dialogue. Behav. Brain Sci. 27, 169–190. 10.1017/S0140525X04000056 15595235

[B17] Prior A. Gollan T. H. (2011). Good language-switchers are good task-switchers: evidence from Spanish–English and Mandarin–English bilinguals. J. Int. Neuropsychol. Soc. 17, 682–691. 10.1017/S1355617711000580 22882810

[B18] Tarlowski A. Wodniecka Z. Marzecová A. (2013). Language switching in the production of phrases. J. Psycholinguist. Res. 42, 103–118. 10.1007/s10936-012-9203-9 22450881

[B19] Toburen T. Meier B. P. (2010). Priming God-related concepts increases anxiety and task persistence. J. Soc. Clin. Psychol. 29, 127–143. 10.1521/jscp.2010.29.2.127

[B20] Verhoef K. M. Roelofs A. Chwilla D. J. (2010). Electrophysiological evidence for endogenous control of attention in switching between languages in overt picture naming. J. Cogn. Neurosci. 22, 1832–1843. 10.1162/jocn.2009.21291 19580393

[B21] Verhoef K. Roelofs A. Chwilla D. J. (2009). Role of inhibition in language switching: evidence from event-related brain potentials in overt picture naming. Cognition 110, 84–99. 10.1016/j.cognition.2008.10.013 19084830

[B22] Yang S. Yang H. (2013). Does bilingual fluency moderate the disruption effect of cultural cues on second-language processing? Proc. Natl. Acad. Sci. U.S.A. 110, E4403. 10.1073/pnas.1316429110 24194553 PMC3839732

[B23] Zhang S. Morris M. W. Cheng C. Y. Yap A. J. (2013). Heritage-culture images disrupt immigrants' second-language processing through triggering first-language interference. Proc. Natl. Acad. Sci. U.S.A. 110, 11272–11277. 10.1073/pnas.1304435110 23776218 PMC3710819

