# Supplementary material for: Iconic Native Culture Cues Inhibit Second Language Production in a Non-immigrant Population: Evidence from Bengali-English Bilinguals
Source: Front Psychol. 2016 Oct 5;7:1516. doi: 10.3389/fpsyg.2016.01516 (PMC5050207; doi:10.3389/fpsyg.2016.01516)
Supplement: Supplementary file 1 [file DataSheet1.docx]

**Appendix 1. Images of six cultural icons considered (out of which two: “Goddess Durga” and “Howrah bridge” were finally used) to be used as background in the naming task.**


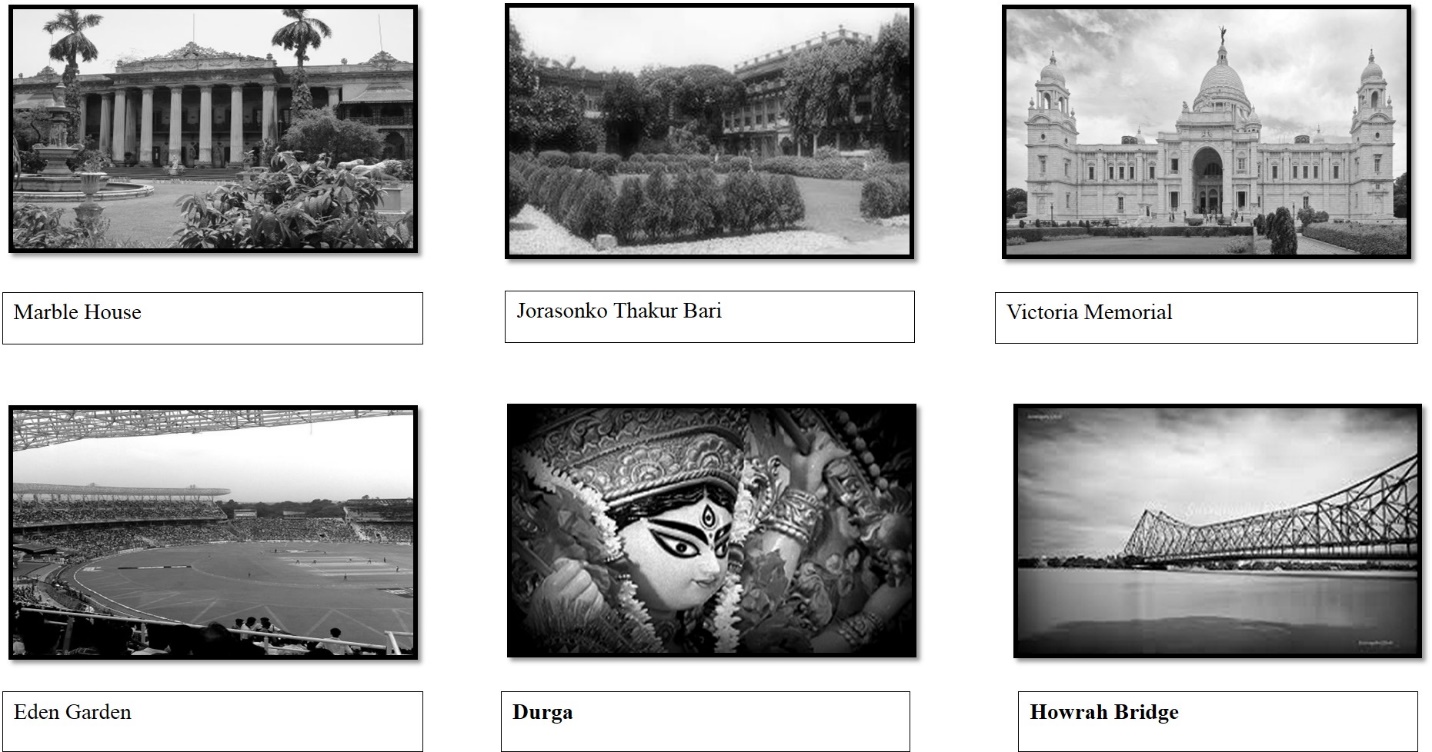


**Appendix 2. Images of neutral cues used in the Experiment.**


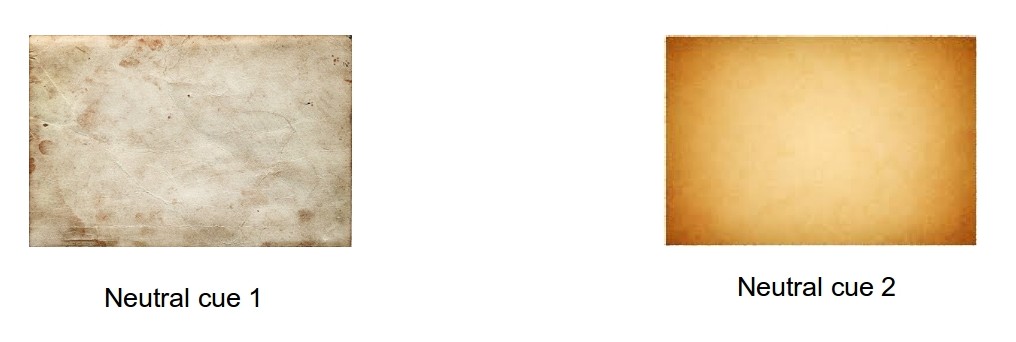


**Appendix 3: Results from statistical analysis.**

| DV: Naming latency | | | | | | | | | |
| --- | --- | --- | --- | --- | --- | --- | --- | --- | --- |
| Effect |  | *F1* | *p* | *η^2^* |  | *F2* | *p* | | *η^2^* |
| Context |  | 0.07 | 0.79 | 0.002 |  | 4.35 | 0.04 | | 0.04 |
| Cue type |  | 0.69 | 0.41 | 0.02 |  | 0.004 | 0.95 | | <0.001 |
| Language |  | 1.65 | 0.21 | 0.04 |  | 4.84 | 0.03 | | 0.05 |
| Context*Cue type |  | 0.64 | 0.43 | 0.02 |  | 0.44 | 0.51 | | 0.01 |
| Cue type*Language |  | 6.44 | 0.01 | 0.14 |  | 4.35 | 0.04 | | 0.04 |
| Context*Language |  | 2.25 | 0.14 | 0.05 |  | 5.9 | 0.02 | | 0.06 |
| Context*Cue type*Language |  | 0.22 | 0.64 | 0.005 |  | 0.02 | 0.89 | | <0.001 |
| DV: Switch cost (Only for “Mixed” context) | | | | | DV: Naming latency^1^ (Blocked + stay trials in Mixed) | | | | |
| Effect |  | *F* | *p* | *η^2^* |  | *F* | *p* | *η^2^* | |
| Language |  | 0.70 | 0.40 | 0.02 |  | 3.03 | 0.09 | 0.07 | |
| Cue type |  | 0.69 | 0.41 | 0.02 |  | 2.24 | 0.14 | 0.05 | |
| Context |  | - | - | - |  | 0.32 | 0.57 | 0.01 | |
| Context*Cue type |  | - | - | - |  | 2.49 | 0.12 | 0.06 | |
| Cue type*Language |  | 2.09 | 0.16 | 0.05 |  | 7.52 | 0.009 | 0.16 | |
| Context*Language |  | - | - | - |  | 0.24 | 0.63 | 0.006 | |
| Context*Cue type*Language |  | - | - | - |  | 0.003 | 0.96 | <0.001 | |

Note: *F1:* Subject-wise effect*, F2:* Item-wise effect; DV: Dependent variable used in repeated measures ANOVA.

^1^ Naming latencies only on stay trials in the “Mixed” context were considered to analyze the cost of mixing languages (referred to as “mixing costs” in the text)
