# Supplementary material for: Iconic Native Culture Cues Inhibit Second Language Production in a Non-immigrant Population: Evidence from Bengali-English Bilinguals
Source: Front Psychol. 2016 Oct 5;7:1516. doi: 10.3389/fpsyg.2016.01516 (PMC5050207; doi:10.3389/fpsyg.2016.01516)
Supplement: Supplementary file 2 [file Image1.PDF]

Supplementary material

Iconic native culture cues inhibit second language production in a non-immigrant population: Evidence from Bengali-English bilinguals

Kesaban Roychoudhuri, Seema Gorur Prasad & Ramesh Kumar Mishra

Correspondence: Ramesh Kumar Mishra, rkmishra@uohyd.ac.in

1 Supplementary figure

All the pictures (200) used in the picture naming task are attached. Each picture is accompanied by its L1 (Bengali) and L2 (English) names.

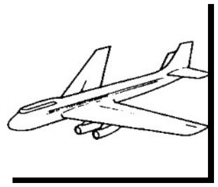

English=Aeroplane

Bengali=Biman

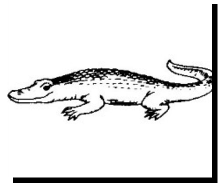

English=Alligator

Bengali=Kumir

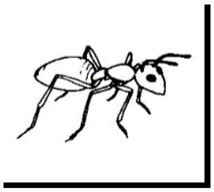

English=Ant

Bengali=Pipra

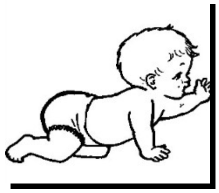

English= Baby

Bengali=Bachcha

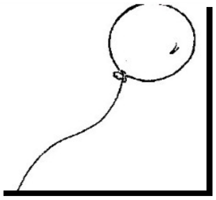

English=Balloon

Bengali=Beluni

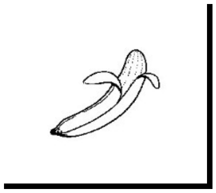

English=Banana

Bengali=Kola

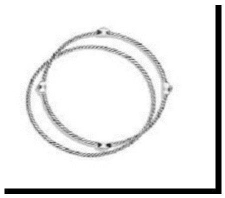

English= Bangle

Bengali=Bala

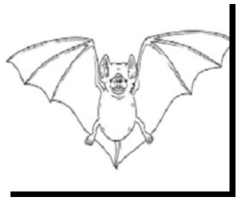

English=Bat

Bengali=Badur

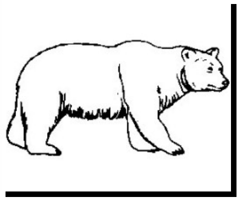

English=Bear

Bengali=Bhalluk

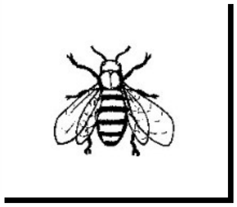

English = Bee

Bengali=Machi

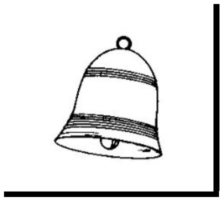

English=Bell

Bengali=Ghonta

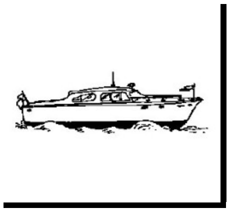

English=Boat

Bengali=Jahaj

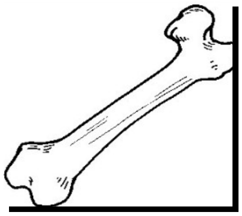

English=Bone

Bengali=Har

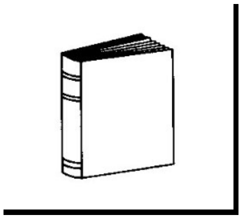

English=Book

Bengali=Boi

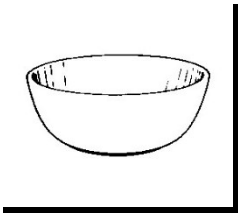

English=Bowl

Bengali=Bati

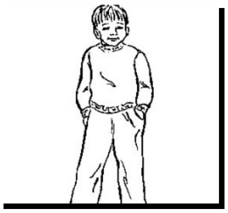

English=Boy

Bengali=Chele

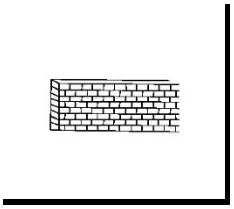

English=Bricks

Bengali=Eet

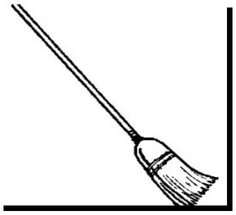

English=Broom

Bengali=Jharu

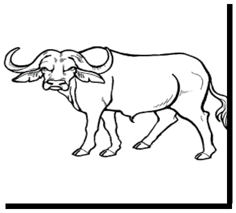

English= buffalo

Bengali= mosh

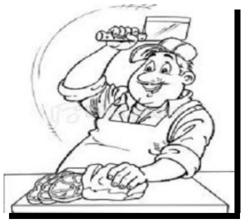

English= Butcher

Bengali= Koshai

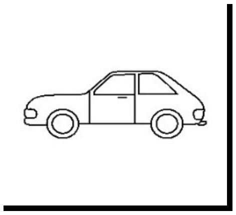

English=Car

Bengali=Gari

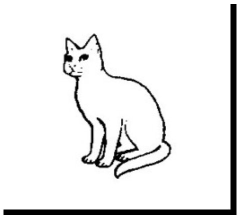

English=Cat

Bengali=Biral

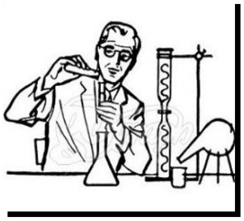

English=Chemist

Bengali=Rosayonbi

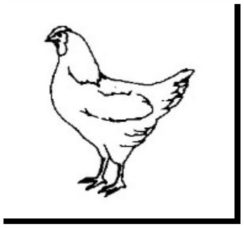

English=Chicken

Bengali=Murgi

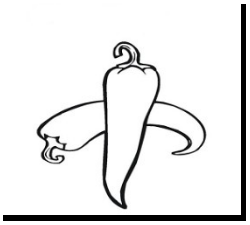

English=Chilli

Bengali=Lonka

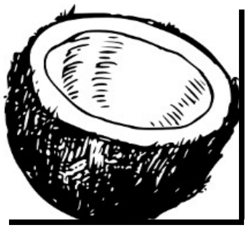

English=Coconut

Bengali=Narkel

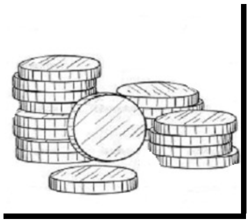

English=coins

Bengali=mudra

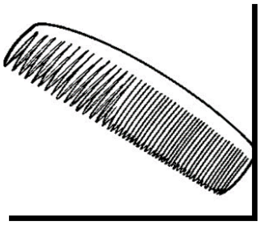

English=Comb

Bengali=Chiruni

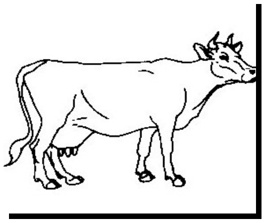

English=Cow

Bengali=Goru

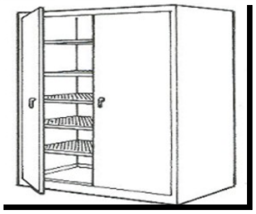

English=Cupboard

Bengali=Almari

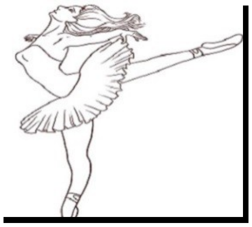

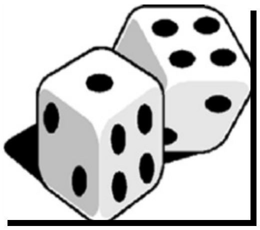

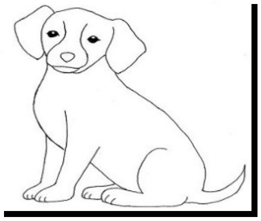

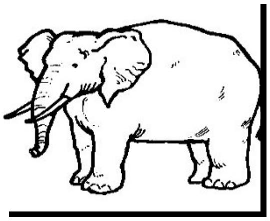

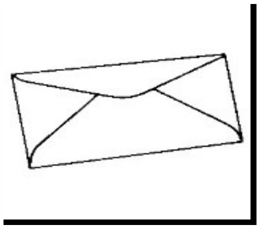

English=Dancer

Bengali=Nortoki

English=Dice

Bengali=Chokka

English=Dog

Bengali=Kukur

English=Elephant

Bengali=Hati

English=Envelop

Bengali=Kham

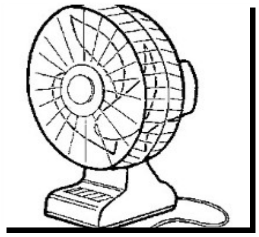

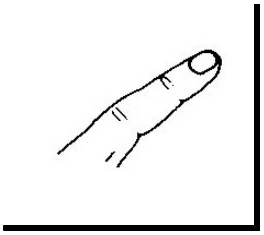

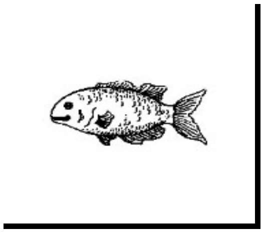

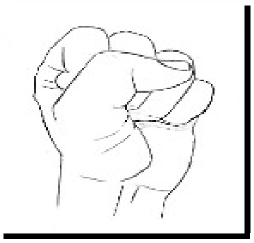

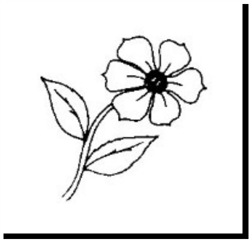

English=Fan

Bengali=Pakha

English=Finger

Bengali=Angul

English=Fish

Bengali=Mach

English=Fist

Bengali=Musthi

English=Flower

Bengali=Phul

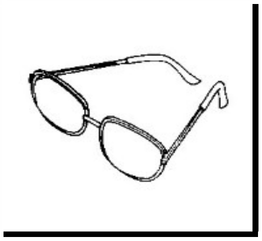

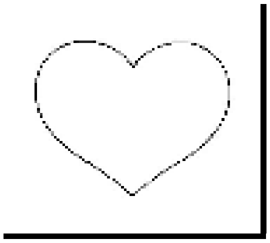

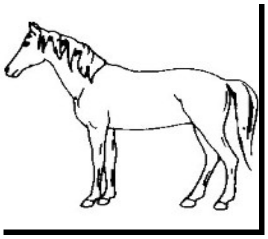

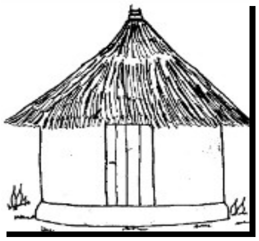

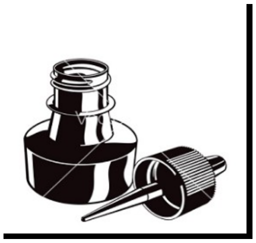

English=Spectacles

Bengali=Chosma

English=Heart

Bengali=Ridoi

English=Horse

Bengali=Ghora

English=Hut

Bengali=Kutir

English=ink

Bengali=kali

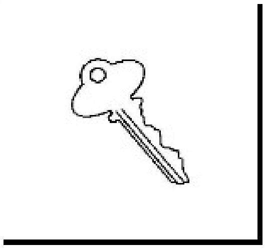

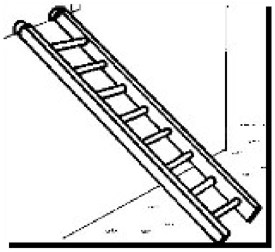

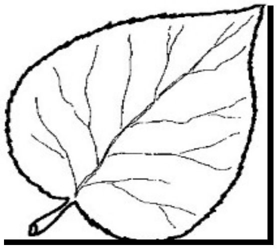

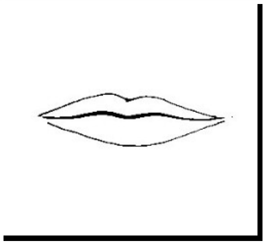

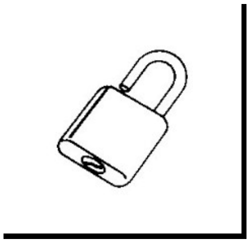

English=Keys

Bengali=Chabi

English=Ladder

Bengali=Siri

English=Leaf

Bengali=Pata

English=Lips

Bengali=Thot

English=Lock

Bengali=Tala

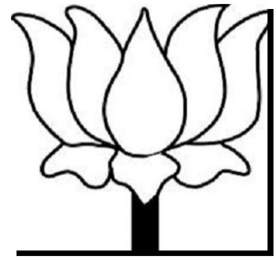

English=Lotus

Bengali=Poddo

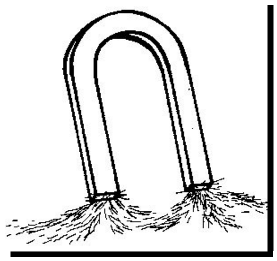

English=Magnet

Bengali=Chumbok

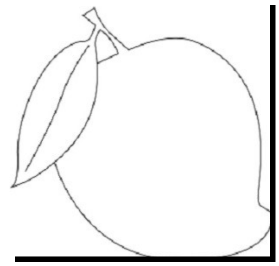

English=Mango

Bengali=Aam

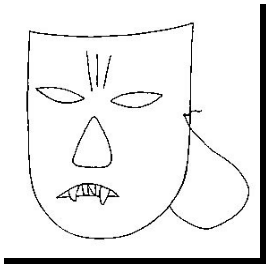

English=Mask

Bengali=Mukhosh

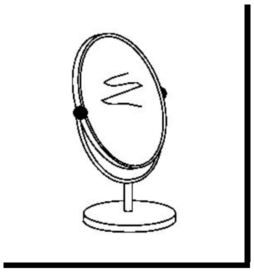

English=Mirror

Bengali=Aina

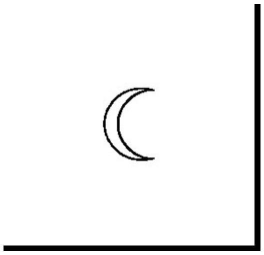

English=Moon

Bengali=Chand

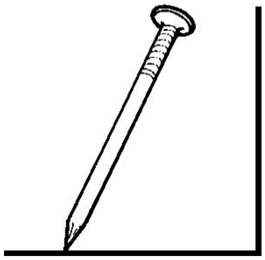

English=Nail

Bengali=Perek

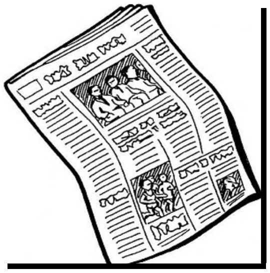

English=Newspaper

Bengali=Potrika

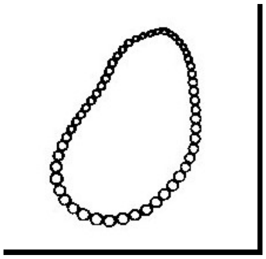

English=Necklace

Bengali=Mala

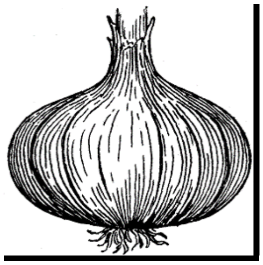

English=Onion

Bengali=Peyaj

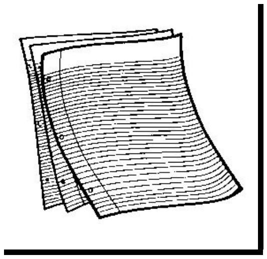

English=Paper

Bengali=Kagoj

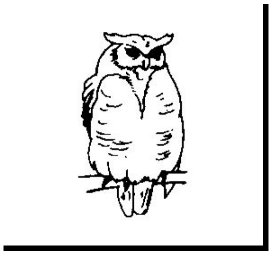

English=Owl

Bengali=Pecha

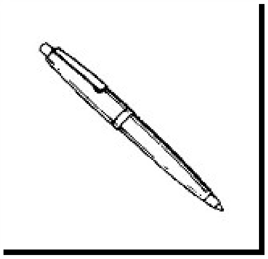

English=Pen

Bengali=Kolom

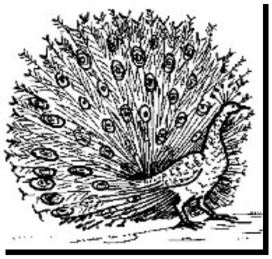

English=Peacock

Bengali=Moyur

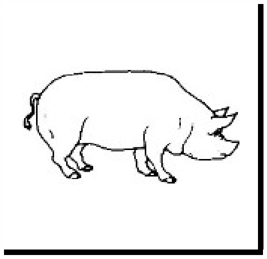

English=Pig

Bengali=Suwor

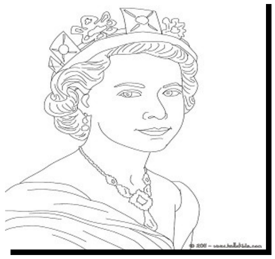

English=Queen

Bengali=Rani

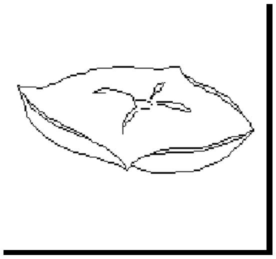

English=Pillow

Bengali=Balish

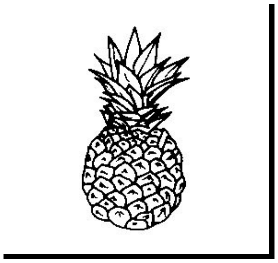

English=Pineapple

Bengali=Anaros

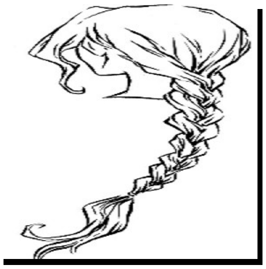

English=Plait

Bengali=Beni

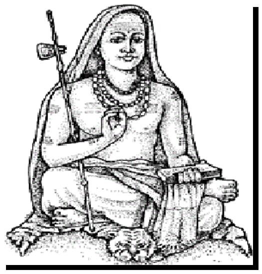

English=Priest

Bengali=Purohit

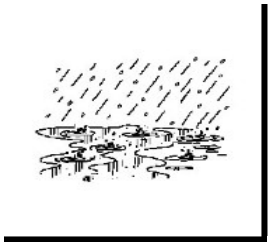

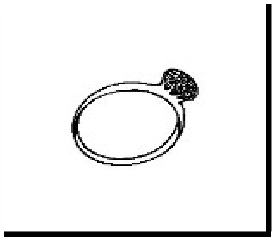

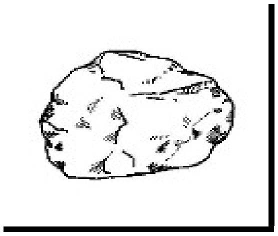

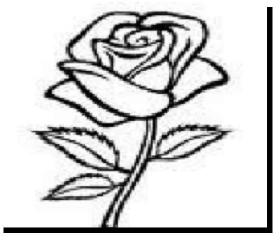

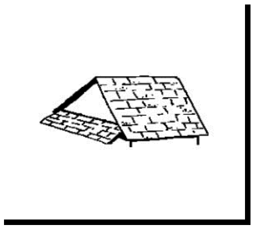

English=Rain

Bengali=Brishti

English=Ring

Bengali=Angti

English=Rock

Bengali=Pathor

English=Rose

Bengali=Golap

English=Roof

Bengali=Chad

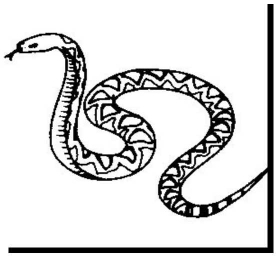

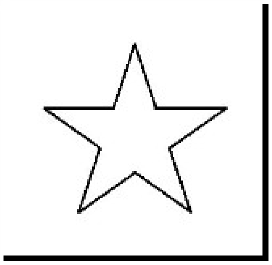

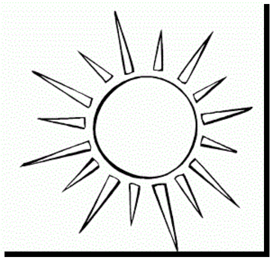

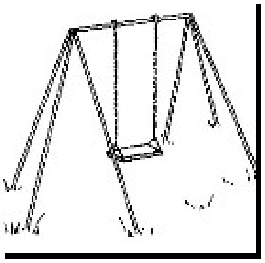

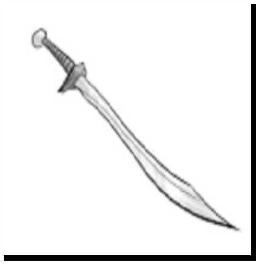

English=Snake

Bengali=Sap

English=Star

Bengali= Tara

English=Sun

Bengali=Surjo

English=Swing

Bengali=Dolna

English=Sword

Bengali=Torowal

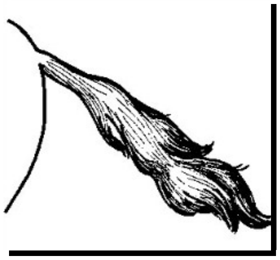

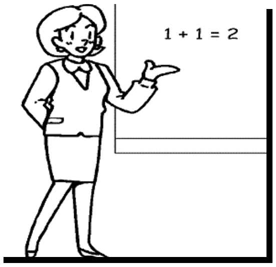

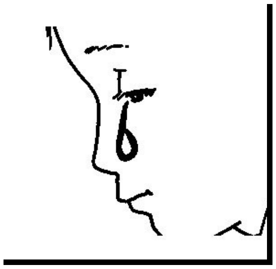

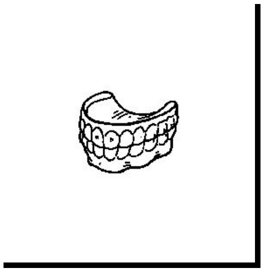

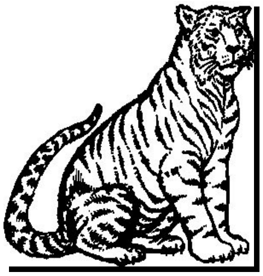

English=Tail

Bengali=Lej

English=Teacher

Bengali=Shikhok

English=Tear

Bengali=Kanna

English=Teeth

Bengali=Dath

English=Tiger

Bengali=Bagh

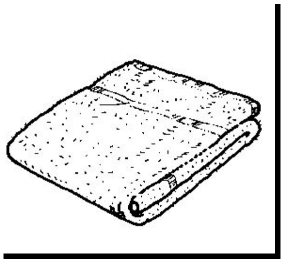

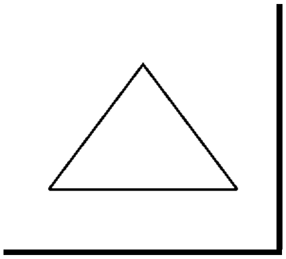

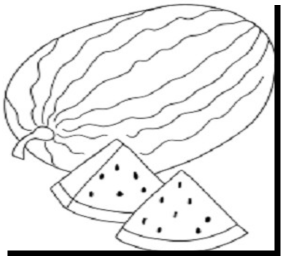

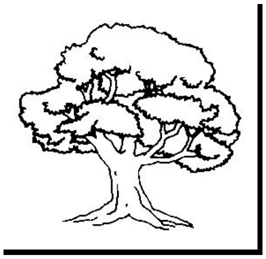

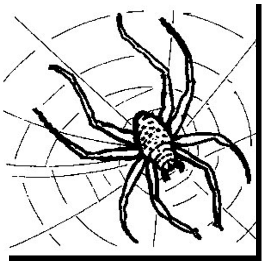

English=Towel

Bengali=Gamcha

English=Triangle

Bengali=Trikon

English=Watermelon

Bengali=Tormujh

English=Tree

Bengali=Gach

English=Spider

Bengali=Makarsha

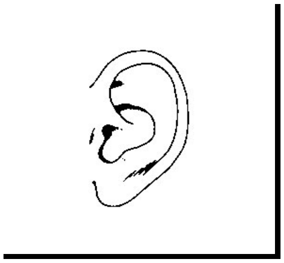

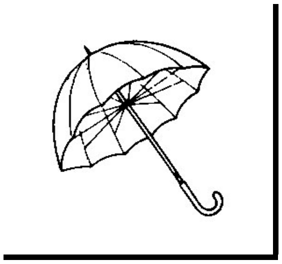

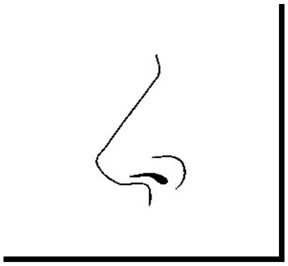

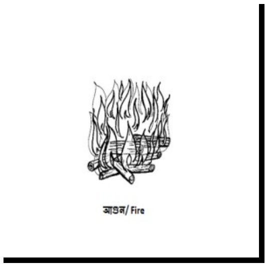

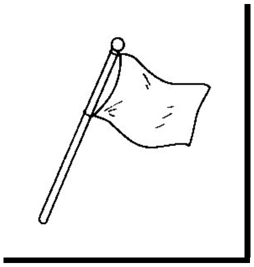

English=Ear

Bengali=Kan

English=Umbrella

Bengali=Chata

English=Nose

Bengali=Nak

English=Fire

Bengali=Aagun

English=Flag

Bengali=Potaka

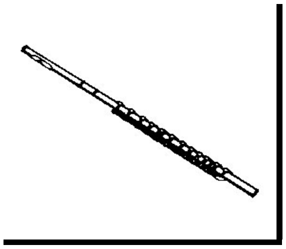

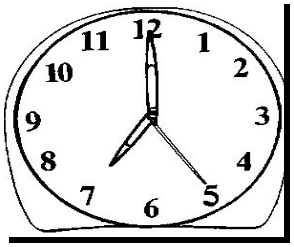

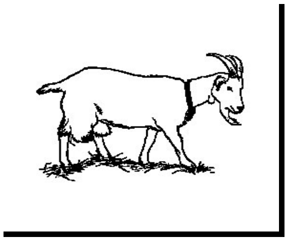

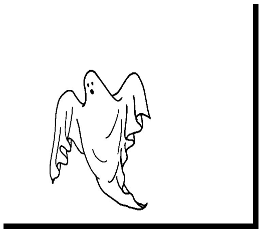

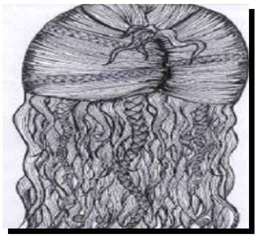

English=Flute

Bengali=Basi

English=Clock

Bengali=Ghori

English=Goat

Bengali=Chagol

English=Ghost

Bengali=Bhoot

English=Hair

Bengali=Chul
